# The Two Oldest English Medical Books

**Published:** 1890-11-08

**Authors:** 


### The Two Oldest English Medical Books.

The first book relating to medicine printed in England was the "Governayle of Helthe," with a separate sheet entitled "Medicina Stomachi," the whole forming a small quarto tract of 36 pages, which was printed by William Caxton about 1491.

Only one copy of this is known to be in existence, viz., that which is in the library of the Earl of Dysart, at Ham House, Surrey; but in 1858 an edition of 55 copies of a reprint was made under the direction of Mr. William Blades, and one of these is now before us.

The author is unknown, one MS. attributing it to be John de Bordeux, and another to Bartholomew, but it is little more than a paraphrase and abridgement of the well-known regimen of the School of Salernum, and is only interesting for its quaint style and orthography. It begins as follows: "In this tretyse that is cleped Governayle of Helthe, what is to be sayd wyth Crystis helpe of Some thynges that longen to bodily helthe, had and to be kept; or to bodily helthe, lost and to be recovered: and is departed in viij chapytures, that is to saye—In the fyrste chapytre, of the profytte of goode Souernayle of helthe. In the ij chapitre, what is first on morow to be don. In the iij chapitre of bodyly exersyce, that is to saye, besynes and his profyte," etc.

The chief points insisted on are: Avoid excess; eat only when hungry; sleep until you wake of your own accord; do not eat raw meat; avoid marshy ground; and be cleanly; all of which is good advice at the present day.

Of much more interest is the second medical work printed in England, which also is in English, and is very rare, not more than half-a-dozen copies being known. This was printed in 1525, "'at London, in Southwarke, by Petrus Treverus," forming a folio of 78 leaves, printed in double columns, with rude woodcuts. The title page is a remarkable one, and is as follows:—

"The noble experyence of the vertuous handyworke of surgeri, practys'd and compyled by the moost experte Mayster Jherome of Bruynswyke, borne in Straesborowe in Almayne, the which hath it fyrst proved, and trewly founde by his owne dayly exercysinge. Item thereafter he hath authorysed, and done it to understande thrugh the trewe sentences of the olde doctours and maysters very experte in the scyence of surgery, as Galienus, Ipocras, Avicenna, Gwydo, Haly Abbas, Sancfrancus of Mylen . . . (*et*) Here also shall ye fynde for to cure and hele all wounded members and other swellynges. Item yf ye fynde ony names of herbes or of other thynges whereof ye have no knowledge, that shall ye knowe playnly by the potecarys. Item here shall you fynde also for to make salves, plasters, powders, oyles, and drynkes for woundes. Item whose desyreth of this science the playne knowledge, let hym oftentymes rede this boke and than he shall gette perfyte understandynge of the noble surgery."

The Original was first published in German at Strassburg, in 1497, with a much shorter title, viz., "Dis ist das buch der Ciruegia, Hand wieckung der wund artzney." The library of the British Museum contains both the original and the translation, the latter being slightly mutilated.

Hieronymus Brannschweig, or Jerome of Brunswick, was a surgeon and apothecary of Strassburg, who was born about 1430, and died about an hundred years later. He was one of the small group of Alsatian physicians and surgeons who were the first to abandon the use of Latin and to publish their works in their mother tongue. And his book is the first published work in which we find reference made to gunshot wounds, although such wounds are spoken of in the manuscript of Pfolzprundt, which dates about 1460, but was not published until 1868.

The first section of Brannschweig's book is devoted to the anatomy of the human body, and is illustrated with rude wood cuts, the most interesting of which is a picture of the head on which is indicated a rough localisation of functions of different parts of the brain. He says "The brayne hath iiij cellys or chambers somewhat longe, and eche celle hath ij partis, and in every parte is a parte of our understandynge. In the fyrst celle is our common wyttes (the anterior part containing the centres of special sense for vision, audition, smell, etc.),. . In the second is the ymaginasyon, in the iij is wynynge and reson, in the iiij is remembrance and memory."

After the anatomy, which, he says, is given in order that the surgeon may know how he should break, dismember, and join again together, the rest of the book is devoted to wounds, fractures, and dislocations, and to formulas for various plasters, oils, and salves. One section is devoted to wounds made with poisoned arrows and to poisoned bites, in which he tells that he saw a man who was bitten in the thumb by another man, with the result that "the thome was so sore it must be cut of, and after that was the hande cut of, and after that was cut of the arme, and the body was swollen so byg that they cowde skante save his lyf."

The next chapter is "Of woundis shot with a gonne where as the venym of the powder abydyth in," and begins as follows: "Also as ony body is shotten with a gon where as the pellet is taken out of the wounde yet in the wounde abydeth the venym of the powder, be it in the arme or leg, wheresoever it be, so thrust a lytyll rope of here through the wounde about all sydes of the wounde, and therwith shal ye drawe out that venym of the powder that is in the wounde." A tent of bacon dipped in ear wax is also recommended to counteract the poison.

The book is in the main a compilation from Avisenna, Roland, Guy du Chanliac, and the four masters, and is interesting chiefly for its quaint phraseology, rather than for any practical therapeutics to be derived from it.

The prologue shows that human nature was much the same four hundred years ago that it is to-day. He advises the young surgeon to call a master in consultation in difficult cases, and to be careful that the doctors do not disagree in the presence of the patient. He says, "In lyke wyse, whan any of you be present alone with your pacyent, blame nat the other that is absente nor dyffame him. But what ye have to saye withe eche other let that be secrete within yourselfe for grewynge of your pacyente, for it myght tourne hym to greate payne and hyndraunce in hys dyseas through your dyscourse. The one shall folow the others counsell, and ye shall hyde nothynge from eche other . . . . Also ye shall not prayse yourselfe, nor blame none other."

